# Metal Complexes for Two‐Photon Photodynamic Therapy: A Cyclometallated Iridium Complex Induces Two‐Photon Photosensitization of Cancer Cells under Near‐IR Light

**DOI:** 10.1002/chem.201604792

**Published:** 2016-11-02

**Authors:** Luke K. McKenzie, Igor V. Sazanovich, Elizabeth Baggaley, Mickaële Bonneau, Véronique Guerchais, J. A. Gareth Williams, Julia A. Weinstein, Helen E. Bryant

**Affiliations:** ^1^ Department of Oncology and MetabolismUniversity of SheffieldSheffieldS10 2RXUK; ^2^ Department of ChemistryUniversity of SheffieldSheffieldS3 7HFUK; ^3^ Department of chemistryDurham UniversityDurhamDH1 3LEUK; ^4^ UMR CNRS 6226Université de Rennes 1, Institut des Sciences Chimiques de Rennes, Campus de Beaulieu35042RennesFrance; ^5^ Harwell Science CampusOX11 0QXUK

**Keywords:** cancer therapy, iridium, singlet oxygen, transition metals, two-photon photodynamic therapy

## Abstract

Photodynamic therapy (PDT) uses photosensitizers (PS) which only become cytotoxic upon light‐irradiation. Transition‐metal complexes are highly promising PS due to long excited‐state lifetimes, and high photo‐stabilities. However, these complexes usually absorb higher‐energy UV/Vis light, whereas the optimal tissue transparency is in the lower‐energy NIR region. Two‐photon excitation (TPE) can overcome this dichotomy, with simultaneous absorption of two lower‐energy NIR‐photons populating the same PS‐active excited state as one higher‐energy photon. We introduce two low‐molecular weight, long‐lived and photo‐stable iridium complexes of the [Ir(N^C)_2_(N^N)]^+^ family with high TP‐absorption, which localise to mitochondria and lysosomal structures in live cells. The compounds are efficient PS under 1‐photon irradiation (405 nm) resulting in apoptotic cell death in diverse cancer cell lines at low light doses (3.6 J cm^−2^), low concentrations, and photo‐indexes greater than 555. Remarkably **1** also displays high PS activity killing cancer cells under NIR two‐photon excitation (760 nm), which along with its photo‐stability indicates potential future clinical application.

Photodynamic therapy (PDT) is a light‐activated treatment offering reduced side effects compared to traditional therapy.^1^ The PDT agent, a photosensitizer (PS), is only activated upon targeted irradiation by light of a PS‐specific wavelength which promotes the PS to its excited, high‐energy state (*PS).

In oxygen‐dependent PDT, cellular oxygen and *PS interactions allow excited‐state energy transfer, regenerating the ground state of the PS and producing reactive oxygen species (ROS) including singlet oxygen (^1^O_2_), with subsequent reactions with the intracellular components leading to cell death. Targeted intracellular localisation of the PS is important for maximum effect with organelles situated nearest to *PS being the most affected.^2^

The photo‐physical properties of many transition metal complexes make them ideal PS candidates. Their key advantage over organic molecules is the heavy atom effect which favours fast singlet to triplet intersystem crossing (ISC). The longer lifetimes, which result from ISC, lead to high yields of ^1^O_2_ and/or other ROS. The ease of chemical modification and photo‐stability adds to the appeal of these complexes. Accordingly, an increasing number of transition metal complexes have been investigated for use in PDT including those of Pt^IV^, Pt^II^, Ru^II^, Re^I^,^3^ and Ir^III^.^4^

One limitation to the clinical use of metal complexes investigated for PDT has been their absorption of light in the UV/Vis region, as the optimal tissue penetration window is 700–900 nm. Two‐photon excitation (TPE), or two‐photon PDT (TP‐PDT) can overcome this barrier. Compounds with high PDT activity under one‐photon excitation at a particular wavelength in the UV/Vis region, should theoretically show PDT‐activity under TPE in the low‐energy NIR region, with the simultaneous advantages of the range of relative tissue transparency, increased potential depth of tissue penetration, and increased spatial targeting.^5^ TP‐PDT requires high two‐photon absorption cross‐section and exceptional photo‐stability ruling out current clinical photosensitizers. A number transition metal complexes3c, 6 have been developed as two‐photon agents, some of which have been shown to induce TPE cell killing in vitro using cell cultures.3c, 6a, 7

We present herein two low‐molecular‐weight, mitochondrial and lysosomal targeting, iridium complexes which display good PS activity under one‐photon excitation in a number of cancer cell lines. Remarkably, one of the complexes is also an efficient PS inducing cell death under TPE, and thus displays highly promising results for TP‐PDT.

The new iridium complexes **1** and **2** (Figure 1) are members of the [Ir(N^C)_2_(N^N)]^+^ family and closely related to the complex [Ir(ppy)_2_(pybzH)]^+^.^8^ **1** and **2** feature bisbenzimidazole and its *N,N*‐dimethylated derivative, respectively, as the N^N ligand (Figure 1). The complexes were prepared as their hexafluorophosphate salts from the chloro‐bridged dimer [Ir(ppy)_2_(μ‐Cl)]_2_, by reaction with 2,2′‐bisbenzimidazole or 1,1′‐dimethyl‐2,2′‐bisbenzimidazole (for **1** and **2**, respectively), (Supporting Information, Figures S3–S5). The absorption spectra of **1** and **2** show moderately intense absorption bands in the visible region due to MLCT transitions (Supporting Information, Figure S2). The emission quantum yields are 0.33 and 0.24, respectively, Emission lifetimes of the order of a microsecond indicate the triplet nature of the emissive state. Importantly, the complexes phosphoresce intensely also under NIR TPE; and **1** has an appreciable two‐photon cross‐section of 112 GM at 760 nm (Supporting Information, Figure S2).

**Figure 1:**
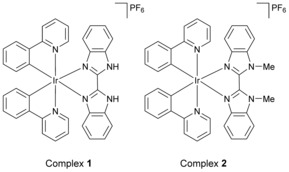
Complexes **1** and **2**.

The TPE phosphorescence imaging of **1** and **2** demonstrate cellular uptake (Supporting Information, Figure S3) with cytoplasmic localisation, similar to that of the previously reported biscyclometallated iridium complexes.4b, 4d Co‐localisation experiments with organelle‐specific fluorescent dyes demonstrate mitochondrial and lysosomal localisation. Within 4 h of exposure, mitochondrial localisation is observed with a Pearson's correlation coefficient *r*=0.547 (Figure 2 A and Supporting Information, Figure S4). Additionally, from 2 h increasing lysosomal staining occurs. By 24 h the lysosomal localisation of **1** becomes dominant with Pearson's correlation coefficients of *r*=0.387 compared to 0.19 for the mitochondrial staining (Figure 2 B and Supporting Information, Figure S4).

**Figure 2:**
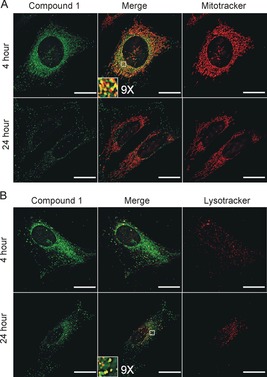
Subcellular localisation of **1**. U2OS cells following 4 or 24 hour incubation with **1** (green), co‐localised with: A) Mitotracker orange (red), or B) Lysotracker (red). Zoomed sections are shown as insets. Scale bars=20 μm.

Cellular uptake was not observed at 4 °C indicating that **1** is actively taken up rather than entering cells by passive diffusion (Supporting Information, Figure S5). A number of cellular uptake pathways were inhibited to determine the uptake route (Supporting Information, Figure S5). Only valinomycin, which is known to cause an increase in the membrane potential of cells, had an inhibitory effect on the uptake of **1** (Supporting Information, Figure S5). Thus uptake of **1** appears to largely occur via a cell membrane potential‐dependent pathway which is consistent with it being a singly‐charged cation.

Clonogenic survival assays in the cervical cancer cell line, HeLa demonstrate that **1** and **2** show high light cytotoxicity giving LD_50_ values of 0.3 and 0.5 μm respectively (405 nm, 3.6 J cm^−2^; Figure 3 and Supporting Information, Figure S6). Complex **1** shows low dark cytotoxicity, with an LD_50_ of >100 μm, whereas **2** shows a much higher dark cytotoxicity, LD_50_=6.2 μm. Comparative PDT activity of a compound can be estimated by the value of the photo‐toxicity index (PI), PI=LD_50_^dark^/LD_50_^light^. The lowest estimated PI for **1** in HeLa cells is >333, but only 12.4 for **2** due to its high dark cytotoxicity, hence **2** was not investigated further. The increased dark toxicity of **2** compared to **1** is likely due to the presence of N−Me groups, which would rule out H‐bonding and profoundly affect intermolecular interactions within the cells. **1** also shows photosensitizing effects in a number of cancer cell lines (Supporting Information, Figure S6), demonstrating the potential broad applicability of **1** for PDT.

**Figure 3:**
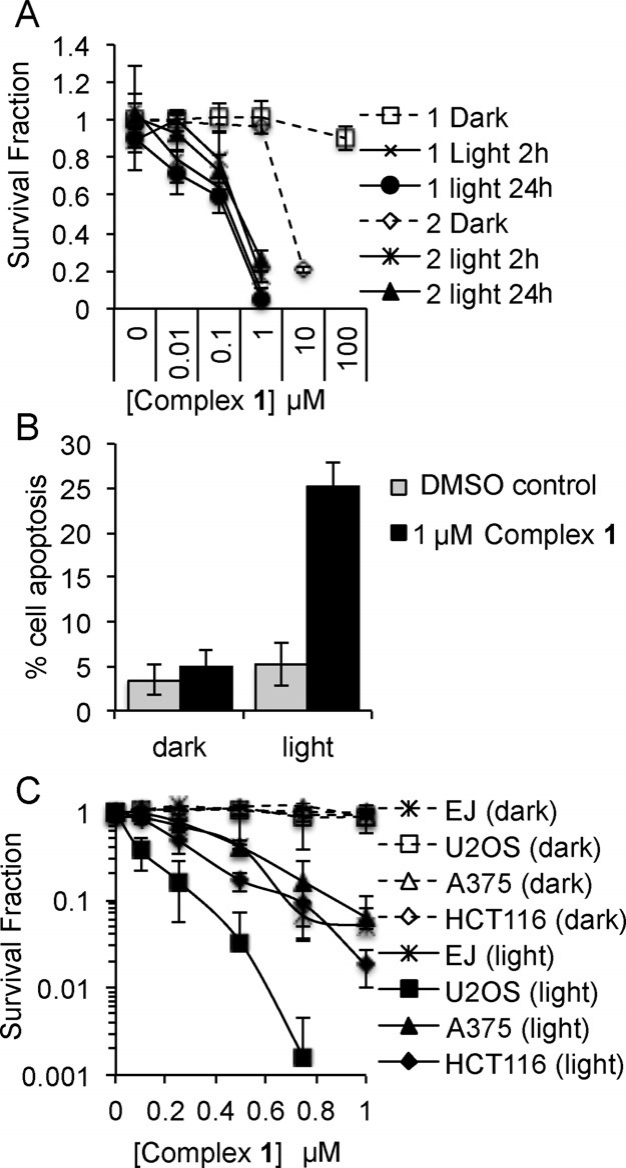
One‐photon‐induced PS activities of complexes **1** and **2**. A) Survival of HeLa cells pre‐incubated for 2 or 24 h with complexes **1** or **2** +/− light. B) Anexin V staining in HeLa cells. C) Survival of bladder (EJ), osteosarcoma (U2OS), melanoma (A375) and colorectal (HCT116) cancer cells 1 +/− light treatment.

To evaluate the importance of oxygen in cell killing by **1** and **2**, the yield of singlet oxygen generation was measured. In air‐equilibrated dichloromethane, high *Φ*(^1^O_2_) of 42 % and 40 % for **1** and **2** respectively were determined directly from the emission of the ^1^Δ_g_ state of O_2_ in the NIR (*λ*_em_ 1275 nm) under 355 nm irradiation, demonstrating the ability of the complex to generate singlet oxygen. In a cellular environment, singlet oxygen immediately reacts with the surroundings, from this point of view, the detection of ROS in cells is an important measurement as it confirms an increase of cellular ROS concentration in the presence of the complex. Here, an approximate 8‐fold increase in ROS was detected in cells treated with **1** and light (405 nm) compared to cells treated with light alone (Supporting Information, Figure S7). Efficient intracellular ROS production and ^1^O_2_ production in solution are suggestive of an oxygen dependant mechanism of cell killing.

The mechanism of cell death was determined to be apoptosis (Supporting Information, Figure S6). It is proposed that apoptotic cell death after light treatment is associated with localisation of photosensitizers to both the mitochondria and the lysosomes.^9^ Here, similar photosensitizing efficacy is seen when the cells were exposed to light following a 2 h incubation—after which mainly mitochondrial localisation is seen, or a 24 h incubation—after which mainly lysosomal localisation is seen (Figure 3). We therefore suggest that light induced cell death by **1** may be due to disruption of one or both of these organelles, which can trigger apoptotic cell death.

The photosensitizing activity of **1** under TPE with 760 nm light was investigated in HeLa cells (Figure 4). The resulting images show apoptosis (green) and cell death (red) induced by TPE photosensitization. The **1**‐exposed cells within the irradiated square clearly show apoptosis/cell death, whereas the surrounding non‐irradiated cells do not. Little to no killing of cells is seen in the absence of **1** with light power as high as 25 mW which under these conditions relates to a dose of 2720 J cm^−2^, this is the lower end of light doses reported to date for two‐photon photosensitizers.7a–7c This result therefore demonstrates the potential of **1** as a specific two‐photon‐activated PS at low concentrations and at doses of light that, alone, are not harmful to cells.

**Figure 4:**
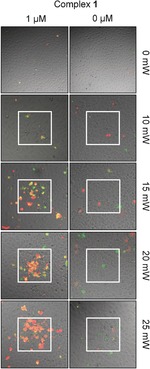
Two‐photon‐induced PS activity of **1**. HeLa cells treated with **1** or DMSO 2 h before irradiation of a central square with 760 nm multiphoton laser. After 24 h cells were stained with markers for apoptosis (Annexin V, red) and cell death (propidium iodide, green). All images are 450×450 μm except those in the 0 mW column which are 900×900 μm.

In summary, we introduce two novel small‐molecule Ir^III^ complexes, which are cell‐permeable, easy to synthesize, possess long‐lived triplet excited states, high linear absorption cross sections in the visible range, and high two‐photon absorption cross sections in the NIR range of the spectrum. **1** has low dark toxicity and is rapidly and actively taken up into a diverse range of cancer cell types, in which localisation is primarily in the mitochondria and lysosomes. High PS activity of **1** has been demonstrated in a number of cancer cell lines under one‐photon excitation with 405 nm light, with an impressive PI index of >333 and up to 555 depending on cell line (lower limit estimate). Remarkably, **1** is also active in photosensitizing cell death by apoptosis under NIR TPE (760 nm), at low concentrations and light doses.

Overall, the results demonstrate the exciting potential of **1** as a future two‐photon PDT agent, and illuminate the potential of Ir^III^ complexes as TP‐PDT agents.

## Supporting information

As a service to our authors and readers, this journal provides supporting information supplied by the authors. Such materials are peer reviewed and may be re‐organized for online delivery, but are not copy‐edited or typeset. Technical support issues arising from supporting information (other than missing files) should be addressed to the authors.

Supplementary
