# Supplementary material for: Metal Complexes for Two‐Photon Photodynamic Therapy: A Cyclometallated Iridium Complex Induces Two‐Photon Photosensitization of Cancer Cells under Near‐IR Light
Source: Chemistry. 2016 Nov 2;23(2):234–8. doi: 10.1002/chem.201604792 (PMC5248616; doi:10.1002/chem.201604792)
Supplement: Supplementary file 1 — Supplementary [file CHEM-23-234-s001.pdf]

CHEMISTRY
A European Journal

Supporting Information

Metal Complexes for Two-Photon Photodynamic Therapy: A Cyclometallated Iridium Complex Induces Two-Photon Photosensitization of Cancer Cells under Near-IR Light

Luke K. McKenzie,[a, b] Igor V. Sazanovich,[a, b, e] Elizabeth Baggaley,[b] Mickaële Bonneau,[c, d] Véronique Guerchais,[d] J. A. Gareth Williams,[c] Julia A. Weinstein,*[b] and Helen E. Bryant*[a]

chem_201604792_sm_miscellaneous_information.pdf

Contents

Experimental Section 3

Materials 3

Synthesis of complexes 3

Photophysical analysis 5

Two photon absorption cross section measurement 6

Cell culture 7

Luminescence imaging 7

Colocalisation studies 8

MitoTracker® Orange 8

Lysotracker® Red 8

Cold uptake assay 9

Inhibitor assay[4] 9

Photodynamic activity assay (one-photon) 10

Dark toxicity assay - MTT 10

Singlet oxygen measurements 11

Reactive oxygen species detection 12

Apoptosis/cell death assay 12

Photodynamic activity assay (two-photon) 12

Table S1. Photophysical data for complexes 1 and 2 14

Supplementary figures 15

Figure S1 ^{1}H NMR spectra of complexes 1 and 2 15

Figure S2. Absorption and emission spectra of complexes 1 and 2. 16

Figure S3. Two-photon emission imaging of 1 and 2 in HeLa cells. Absorption and emission spectra of complexes 1 and 2 16

Figure S4. Time dependent co-localisation of complex 1 with mitochondria and lysosomes.. 18

Figure S5. Active uptake of complex 1 by cells.. 20

Figure S6. One-photon-induced PS activity of complexes 1 and 2. 21

Figure S7. Light induced intracellular reactive oxygen species (ROS) generation by complex 1. 22

References 23

Experimental Section

Materials

All chemicals were purchased from Sigma-Aldrich Company Ltd. Dorset, England unless otherwise stated. Stock solutions of complexes **1** and **2** were made up in dimethyl sulfoxide, DMSO, and stored at −20°C.

Synthesis of complexes

The iridium precursor $[Ir(ppy)_2(\mu\text{-}Cl)]_2$ was prepared from $IrCl_3.3H_2O$ and 2-phenylpyridine using standard methodology[1]. ^{1}H and ^{13}C{^{1}H} NMR spectra were recorded at the frequencies indicated on Varian 600 MHz or 700 MHz instruments. Chemical shift data are in ppm; spectra are referenced using residual solvent ^{1}H and ^{13}C shifts. ^{1}H coupling constants *J* are reported in Hz to the nearest 0.5 Hz. Scans of the ^{1}H NMR spectra of complexes **1** and **2** are provided in the Supplementary Information. High resolution mass spectrometry was carried out using electrospray ionisation on a Waters LCT Premier XE or a Thermo-Finnigan LTQ FT mass spectrometer

1,1'-Dimethyl-2,2'-bisbenzimidazole

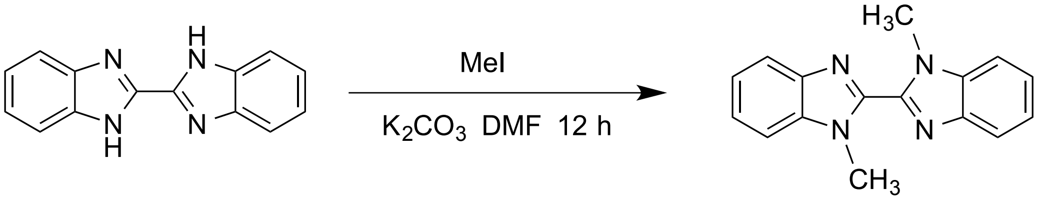

2,2'-Bisbenzimidazole (50 mg, 0.213 mmol) and potassium carbonate (74 mg, 0.533 mmol) were stirred in DMF (5 mL) for 30 min, and iodomethane (181 mg, 1.28 mmol) was then added. The mixture was stirred for 12 h. The beige precipitate that had formed was separated by centrifugation, washed with diethyl ether (3 × 10 mL) and acetone (2 × 10 ml), and dried to give the desired product (42 mg, 75%). M.p. > 250˚C. ^{1}H NMR (600 MHz, d_6-DMSO) δ = 7.81 (2H, d, J = 8.0), 7.72 (2H, d, J =

8.0), 7.41 (2H, t, J = 8.0), 7.34 (2H, t, J = 8.0), 4.27 (6H, s, CH_3). $^{13}C\{^1H\}$ NMR (151 MHz, d_6-DMSO) δ = 142.9, 141.8, 135.9, 123.7, 122.6, 119.6, 110.9, 54.3. HRMS (ES+) *m/z* = 263.1303 ($M+H^+$) calcd for $C_{16}H_{15}N_4$ *m/z* = 263.1297.

$[Ir(ppy)_2(bbzH_2)]^+[PF_6]^-$ Complex 1

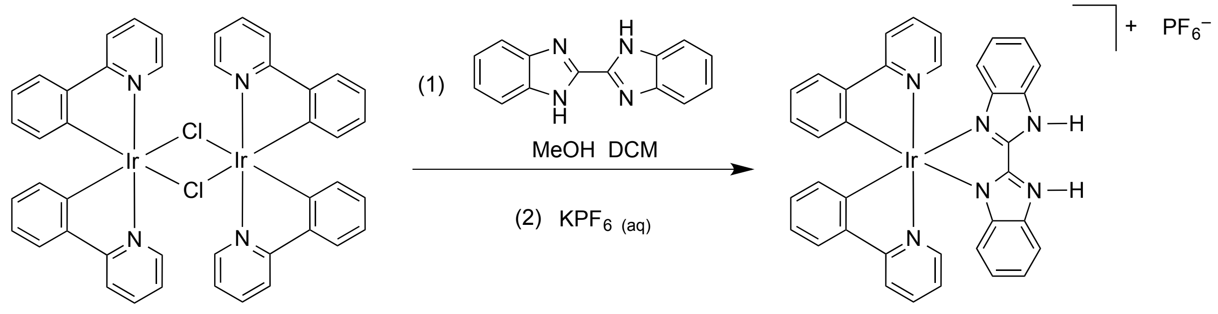

Complex **1**

A suspension of $[Ir(ppy)_2(\mu\text{-}Cl)]_2$ (50 mg, 0.046 mmol) and 2,2'-bisbenzimidazole (25 mg, 0.103 mmol) in a mixture of MeOH and CH_2Cl_2 (1:1) was heated at 52°C for 24 h under a positive pressure of nitrogen gas. After cooling to ambient temperature, the solvent was removed under reduced pressure and the residue was dissolved in the minimum volume of water / acetonitrile (1:1). The resulting solution was added dropwise to a saturated aqueous solution of KPF_6 and the resulting precipitate was collected by centrifuge and washed with water. The yellow product was dried under vacuum (51 mg, 75%). M.p. > 250°C. ^1H NMR (700 MHz, $CDCl_3$) δ = 7.83 (2H, d, *J* = 8.0), 7.68 (6H, m), 7.65 (2H, t, *J* = 7.0), 7.30 (2H, t, *J* = 7.5), 7.08 (2H, t, *J* = 7.5), 6.99 (2H, t, *J* = 7.5), 6.94 (2H, t, *J* = 7.5), 6.86 (2H, t, *J* = 7.0), 6.51 (2H, d, *J* = 7.5), 6.19 (2H, d, *J* = 8.0). HRMS (m/z): [M^+] calcd for $C_{36}H_{26}N_6{}^{191}Ir$, 733.1825; found 733.1846. Elemental analysis calcd (%) for $C_{36}H_{26}N_6IrPF_6$ C 49.2, H 3.0, N 9.6; found: C 50.1, H 3.3, N 10.0. The presence of a small proportion of the deprotonated (*i.e.* singly N-protonated) form of this complex may account for the deviation from the calculated values. ^1H NMR spectrum Figure S1A

$[Ir(ppy)_2(bbzMe_2)]^+[PF_6]^-$ Complex 2

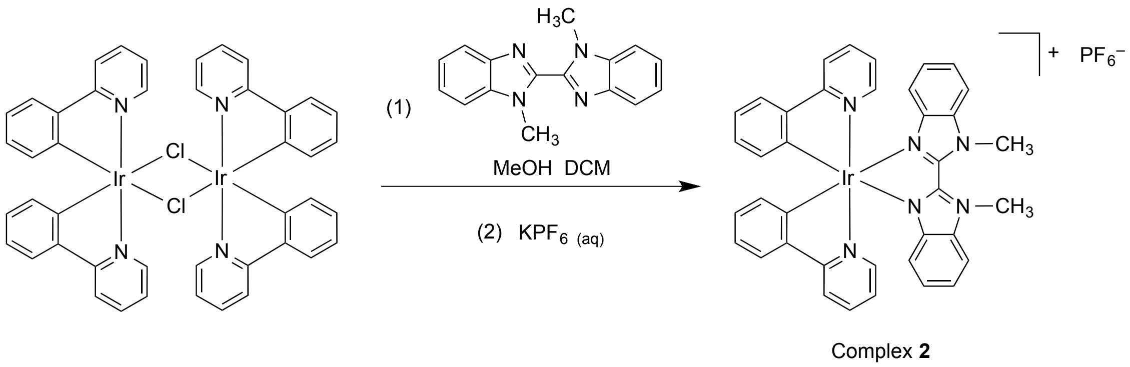

Complex **2** was prepared using a similar procedure to that used for complex **1** from $[Ir(ppy)_2(\mu\text{-}Cl)]_2$ (50 mg, 0.046 mmol), but with 1,1'-dimethyl-2,2'-bisbenzimidazole (27 mg, 0.103 mmol) in place of 2,2'-bisbenzimidazole. The product, a pale yellow powder, was again isolated as the hexafluorophosphate salt (44 mg, 63%). Mp > 250°C. ^1H NMR (700 MHz, $CDCl_3$) δ = 8.03 (2H, d, J = 5.5), 7.78 (2H, d, J = 7.5), 7.63 (4H, overlapping m), 7.56 (2H, d, J = 8.5), 7.38 (2H, t, J = 7.5), 7.07 (2H, t, J = 7.5), 7.00 (4H, overlapping m), 6.92 (2H, t, J = 7.5), 6.40 (2H, d, J = 7.5), 6.23 (2H, d, J = 8.5). HRMS (m/z): $[M^+]$ calcd for $C_{38}H_{30}N_6{}^{191}Ir$ 761.2138, found 761.2109. Elemental analysis calcd (%) for $C_{38}H_{30}N_6IrPF_6$ C 50.3, H 3.3, N 9.3; found: C 50.5, H 3.6, N 8.9. ^1H NMR spectrum Figure S1B

Photophysical analysis

UV-visible absorption spectra in solution were recorded on a Uvikon XS spectrometer in quartz cuvettes of 1 cm pathlength. Luminescence spectra were recorded in quartz cuvettes of the same dimensions, modified with apparatus for connection to a vacuum line. The spectra were obtained on degassed and aerated solutions using a Fluoromax-2 spectrometer; the spectra and data reported are corrected for the wavelength dependence of the emission monochromator and detector (a Hamamatsu R928 photomultiplier tube). Lifetimes were determined by time-correlated single photon counting using Edinburgh Instrument mini-tau following excitation with a pulsed laser diode at 405 nm, 75 ps.

Two photon absorption cross section measurement

The two-photon absorption cross-section of complex **1** was measured at 760 nm in MeCN by a comparative technique, using Fluorescein in NaOH (0.1 M) as the emission standard.

Data collection: Emission spectra were recorded using a bespoke, two-photon laser scanning microscope, constructed in the Central Laser Facility of the Rutherford Appleton laboratory. Solutions under investigation (1.0×10-4 M) were transferred (one at a time) to a large glass cover slip and placed into the sample holder of the laser scanning microscope. The emission signal was recorded from a specific pixel position (in the center of the 256x256 frame) using an Acton 275 spectrograph and a CCD (Andor iDUS). The spectra were recorded across a range of lasers powers until the saturation limit of the sample was reached. Emission spectra from the reference solutions were measured immediately after the sample under identical conditions.

Data processing: Emission intensity (F) for sample and reference was integrated over a 5 nm window (typically 18 data points) where the overlap and gradient of emission curves were comparable. The linear relationship of F vs. W^2 (emission intensity vs. laser power squared) was determined for the 5 nm window and the gradient (b) obtained by applying a linear fit. The gradient (b) was then used to calculate the two-photon absorption cross-section according equation (1) reported in [2].

$$\sigma_s = \sigma_r b_s c_r \varphi_r / (b_r c_s \varphi_s) \qquad (1)$$

Where σ is the two-photon absorption cross-section, b is the slope of linear dependence of F vs. W^2, c is the molar concentration, and φ is the differential emission quantum yield in the 5nm spectral range selected for comparison. The subscript s or r means either sample or reference. The value σ_r for Fluorescein was taken as 45 GM at 760 nm.

The relative quantum yield φ was obtained on a Jobin-Yvon Fluoromax 4 fluorimeter under one-photon excitation. The total emission quantum yield was measured for

aerated solutions of complex **1** in the appropriate solvents, using $[Ru(bipy_{)3}]Cl_2$ (ϕ = 0.028[3]) as the reference standard. The relative emission quantum yield φ was calculated by multiplying the total quantum yield by the fraction of emission intensity in the 5 nm range previously selected, with the respect to the intensity of entire emission spectrum.

Cell culture

The cell lines HeLa (human cervical cancer), HCT116 (colorectal carcinoma), EJ (bladder carcinoma), U2OS (Bone osteosarcoma) and A375 (malignant melanoma) were cultured using Dulbecco's modified Eagles Medium (DMEM) (Lonza, Cambridge UK) with 10% fetal calf serum (FCS) (Lonza, Cambridge UK). All cells were incubated at 37°C under 5% CO_2. All cell lines were purchased from American Type Culture Collection – LGC partnership (Teddington, UK) and used within 20 passages of purchase. Cells were routinely checked for mycoplasma contamination.

Stock solutions of **1** and **2** were stored in DMSO at 5 mM and 0.5 mM at –20°C, aliquotted in small volumes to avoid freeze/thaw cycles.

Luminescence imaging

Cover glasses (22 x 22 mm) were sterilised (industrial methylated spirits, IMS) and placed flat in 6 well plates. Cells were seeded at a density of ~1 × 10^5 cells per well and allowed to adhere overnight in culture media. The iridium complex at the desired concentration was added and incubated for the required time. Cells were washed 3 times in PBS and fixed in 4% paraformaldehyde solution in PBS at 4°C for 20 mins before they were washed again (PBS x 3) and mounted to microscope slides (IMMU-MOUNT™, Life Technologies Ltd, Paisley, UK).

Colocalisation studies

Cover glasses (22 x 22 mm) were sterilised (industrial methylated spirits, IMS) and placed flat in 6 well plates. Cells were seeded at a density of ~1×10^4 cells per well and allowed to adhere overnight in culture media. Complex **1** (1 μM) was added and incubated (2 hours). Co-stains were added as follows:

MitoTracker® Orange

After incubation in **1** for various times a final concentration of 100 nM MitoTracker® orange CMTMRos (Molecular Probes® by Life Technologies Ltd, Paisley, UK) was added and incubated together for 30 mins. Cells were washed three times in PBS and fixed in 4% paraformaldehyde solution in PBS at 4°C for 20 mins before they were washed again (PBS x 3) and mounted to microscope slides (IMMU-MOUNT™, Life Technologies Ltd, Paisley, UK).

Lysotracker® Red

After incubation in **1** for various times a final concentration of 75 nM LysoTracker® Red DND-99 (Molecular Probes® by Life Technologies Ltd, Paisley, UK) was added and incubated together for 2 hours. Cells were washed three times in PBS and fixed in 4% paraformaldehyde solution in PBS at 4°C for 20 mins before they were washed again (PBS x 3) and mounted to microscope slides (IMMU-MOUNT™, Life Technologies Ltd, Paisley, UK).

The slides were imaged by wide-field microscopy (OMX optical microscope (version 4), GE Healthcare) using a 60x lens (Upsalto NA: 1.42, reflective index: 1.514, oil) with image stack deconvolution (z step 0.250 μM, softworx software) and by two-photon microscopy (Coherent Chameleon femto-second pulsed laser, Inverted Zeiss LSM 510 NLO microscope) Multiphoton light (760 nm) was used to excite **1** and **2**; emission was registered in the region 500-550 nm.

Colocalisation indices were calculated using the open source imaging software Fiji (based on imagej) and the coloc 2 colocalisation tool. The threshold regression chosen was Bisection.

Cold uptake assay

Cover glasses (22 x 22 mm) were sterilised (industrial methylated spirits, IMS) and placed flat in two 6 well plates. Cells were seeded at a density of ~1 × 10^4 cells per well and allowed to adhere overnight in culture media. Prior to treatment, one 6-well plate was refrigerated at 4°C for 20 mins whilst the other remained in the incubator (37°C, 5% CO_2). Media was removed and cells treated with cold (4°C) or warm (37°C) DMEM containing 5 μM **1**, and replaced in refrigerator or incubator respectively for 30 min. Following treatment cells were washed three times in PBS and fixed in 4% paraformaldehyde solution in PBS at 4°C for 20 mins before they were washed again (PBS x 3) and mounted to microscope slides (IMMU-MOUNT™, Life Technologies Ltd, Paisley, UK). Slides were imaged (Nikon A1 confocal system, 40x oil objective, 405 nm laser) and mean intensity over 100 cells was quantified.

Inhibitor assay[4]

Cover glasses (22 x 22 mm) were sterilised (industrial methylated spirits, IMS) and placed flat in 6 well plates. Cells were seeded at a density of ~1 × 10^4 cells per well and allowed to adhere overnight in culture media. Cells were washed (3 x PBS) and pretreated with inhibitors (50 μM chloroquine for 1 hour or 1 mM tetraethylammonium for 20 mins or 50 μM valinomycin for 30 mins) in culture medium before 5 μM **1** was added for a further 30 mins. Cells were washed three times in PBS and fixed in 4% paraformaldehyde solution in PBS at 4°C for 20 mins before they were washed again (PBS x 3) and mounted to microscope slides (IMMU-MOUNT™, Life Technologies Ltd, Paisley, UK). Slides were imaged (Nikon A1 confocal system, 40x oil objective, 405 nm laser) and mean intensity over 100 cells was quantified.

Photodynamic activity assay (one-photon)

A 6-well plate was seeded at 50000 cells/well and left to incubate overnight. Wells were treated with varying concentration of complex (0-1 μM for 2 hours, max 0.2% DMSO) or equivalent DMSO control in culture medium. Cells were washed three times in PBS and removed from the plates by trypsonization. A volume of 2 ml of clear culture media (DMEM w/o L-glutamine or phenol red) was added to each well. The contents of each well were then equally split between a glass vial (for light irradiation) and a plastic Eppendorf tube (for dark control) and placed on ice. The glass vials were treated with 405 nm light for 3 mins at 20 mW cm^{-2} whilst the Eppendorf tubes were kept in the dark. Once treated, cells were plated for each condition at 1000 and 2000 cells per dish on 100 mm petri dishes in standard culture media. Plates were incubated for 10 - 12 days to form colonies before staining with 4% methylene blue in 70% methanol and counting. Each colony was considered to represent a single surviving cell and survival fraction calculated for each condition compared to no PS and no light.

For clonogenic assays in the dark with 100 μM complex **1**, 6-well plates were seeded with cells at 250 and 500 per well and left overnight. Wells were treated with complex or DMSO control. Following incubation for 2 hours media was replaced. Plates were incubated for 5-10 days until colonies formed and stained as above.

Dark toxicity assay - MTT

96-well plates were seeded with cells at 1000/well and left overnight. Wells were treated with 1-40 μM complex **1** or **2** or DMSO control and incubated for either 2 or 24 hours before replacing the treatment with fresh media. After 5 days further growth, 25 μl of 3mg/ml thiazoyl blue (MTT) solution was added to each well. Following incubation for 3 hours the solution was removed from each well and 250 μl / well DMSO added ensuring mixing of crystals. Optical density of wells at 540 nm was recorded on a plate reader (Multiskan fc, Thermo Fisher Scientific, Warrington, UK).

Singlet oxygen measurements

Due to the short lifetime of singlet oxygen in an aqueous environment, singlet oxygen was detected directly in dichloromethane (DCM) by measurement of singlet oxygen luminescence(λ_{em} ~1275 nm) following photo excitation of complexes **1** and **2** at room temperature in air saturated solutions of DCM[5]. The third harmonic of a Q-SW Nd:YAG (λ = 355 nm, ~8 ns pulse length, laser model Ls-1231M from LOTISII) was used. The time resolved signal of $^1O_2$ luminescence at 1275 nm was detected by a liquid nitrogen cooled InGaAs photodiode of Ø3 mm active area (J22D-M204-R03M-60-1.7, Judson Technologies). The output from the photodiode was coupled into the low-noise current amplifier (DLPCA-200, FEMTO Messtechnik GmbH), the amplifier output signal was recorded with a digital oscilloscope (TDS 3032B Tektronix) and transferred to the computer. To selectively detect the $^1O_2$ emission, the high-contrast bandpass optical filter (1277 nm center wavelength, 28 nm FWHM, custom-made by Izovac, Belarus) was fitted in front of the InGaAs photodiode. To increase the light collection efficiency, the spherical broadband mirror was set behind the sample to reflect the NIR emission through the sample towards the detector.

The optical densities of both the studied compound and the standard were matched at 355 nm, and the same solvent was used for both the studied compound and the standard. The series of experiments was performed at set of different excitation energies ranging from 20 μJ to 500 μJ per pulse. The presented $\varphi(^3O_2)$ values were obtained in low-energy limit, for which the decay of singlet oxygen emission was mono-exponential.

The quantum yield of singlet oxygen production was determined by comparing the slopes of the linear plots of the initial intensity vs. laser energy for the compounds and that of the standard (perinapthenone, $\varphi(^1O_2)$=95%)[6]. Emission lifetime for $^1\Delta_g$ sensitised by the compounds and the standard were similar (within the range 70–90 μs), indicating that $^1\Delta_g$ does not react with the photosensitiser in its ground state.

Reactive oxygen species detection

The generation of reactive oxygen species, ROS, was monitored using the OxiSelect^{TM} Intracellular ROS Assay Kit (Cambridge Bioscience Ltd, Cambridge, UK). Cell culture petri dishes (100 mm) were seeded with 1x10^6 cells per dish and cultured overnight. Complex **1** was added at 0.1 μM for 2 hours before cells were removed from the plates by trypsinization and washed twice in PBS. Cell pellets were re-suspended in 200 μl 1 mM DCFH-DA and incubated at 37°C for 30 mins. Cells were washed and suspended in 2 ml DMEM w/o L-glutamine or phenol red. Cells were then treated with light as in the light toxicity assay and cell pellets were obtained for each condition.

For Flow cytometry analysis, cell pellets were re-suspended in 300 μl PBS and cell solutions were analysed by flow cytometry (BD™ LSR II flow cytometer) with excitation at 480 nm and emission read at 530 nm.

Apoptosis/cell death assay

Cells were plated on cell culture dishes at 1x10^6 cells/plate and incubated overnight. Plates were then incubated with 0.1-1 μM complex **1** or DMSO control for 2 hours before light treatment following the photodynamic activity assay. Following light treatment, cells were washed once with PBS and cell pellets stained for annexin V using Flowcollect® Annexin Red Kit (Millipore (U.K.) Limited, Watford, UK) according to manufactures instructions. Analysis was performed by flow cytometry (BD™ LSR II flow cytometer).

Photodynamic activity assay (two-photon)

A 96-well plate (Ibidi, Thistle Scientific LTD, Glasgow, UK) was seeded and incubated overnight to give a seeding density of ~80%. Wells were treated with 1 μM complex **1** (0.2% DMSO in media) or DMSO control for 2 hours before the media was replaced with PBS. Using a confocal microscope (Zeiss LSM510 NLO Upright)

with a multiphoton laser (Coherent Chameleon femto-second pulsed laser ~120 fs pulse length, 80 MHz repetition rate), patches of cells were treated treated by scanning the focussed laser beam (760 nm) across the rectangular area of the sample (215 x 215 μm, 1024 x 1024 pixels, 6.6 μs pixel dwell time, 8 repeated scans) using microscope scanner and the 96-well plate placed on the microscope sample stage. The laser power at sample used during scans ranged between 10 mW to 25 mW. The treatment areas were recorded. Following laser treatment, PBS was replaced with media and cells left to incubate for 24 hours. Cells were then stained with Annexin V using Annexin V-Alexa Fluor® 488 conjugate, (Life Technologies, Paisley, UK) and propidium iodide for 15 minutes. The cells were washed twice with PBS prior to imaging (excitation with Ar-ion laser at 488 nm, or He/Ne laser at 543 nm, 20x/0.8 dry objective).

Table S1. Photophysical data for complexes 1 and 2

Performed in deoxygenated CH_2Cl_2 at room temperature, except where indicated otherwise. The yellow emission of complex **1** has a maximum at 534 nm, whilst the more orange emission of complex **2** is red-shifted to 593 nm in DCM. The emission quantum yields are 0.33 and 0.24 respectively, whilst the lifetimes are of the order of a microsecond, indicative of the phosphorescent nature of the emission. At 77 K, the spectra are somewhat blue-shifted and display more pronounced vibrational structure, with lifetimes increased to around 4 μs.

	Complex **1**	Complex **2**
Absorption λ_{max} / nm (ε / M^{-1} cm^{-1})	260 (34700), 325 (28600), 339 (31000), 369 (15800), 419 (3540), 468 (900)	272sh (38700), 335 (26100), 347 (26200), 386sh (10500)
Emission λ_{max} / nm	534	593
Φ_{lum} *(a)*	0.33	0.24
τ / μs	1.9	1.0
λ_{max} at 77 K / nm *(b)*	479, 516, 552sh	508, 547, 590
τ at 77 K / μs *(b)*	4.1	4.5

(a) Luminescence quantum yield measured using $[Ru(bpy)_3]Cl_2$ in H_2O as the standard. *(b)* In diethyl ether / isopentane / ethanol (2:2:1 v/v).

Supplementary figures

Figure S1 ^{1}H NMR spectra of complexes 1 and 2

(A) 1H NMR spectrum of complex 1 in CDCl3 at 295 K

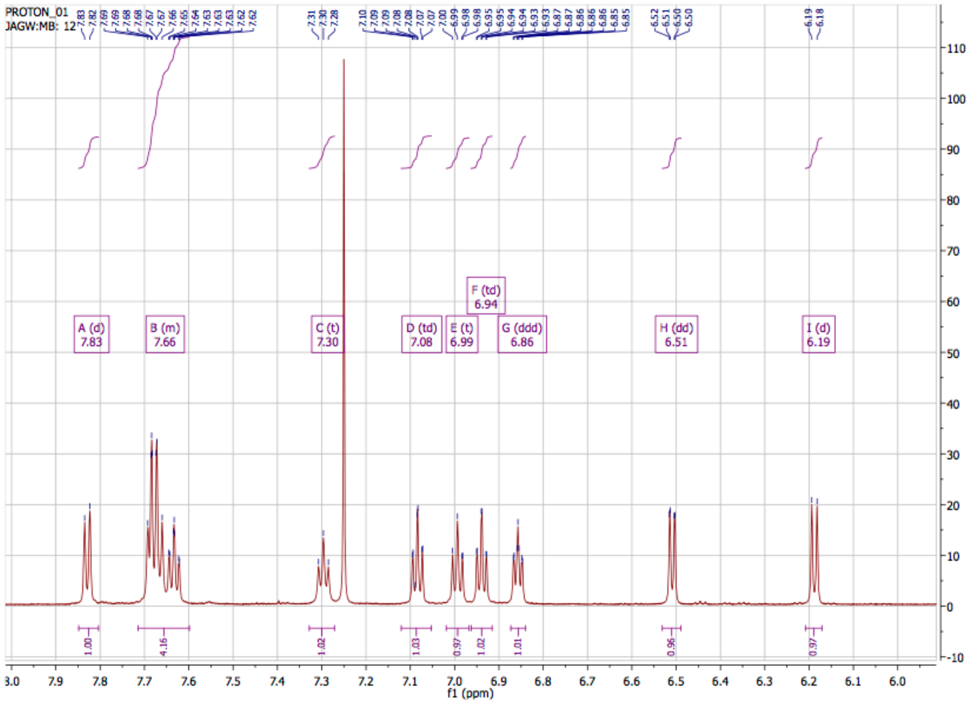

(B) ^{1}H NMR spectrum of complex 2 in $CDCl_3$ at 295 K

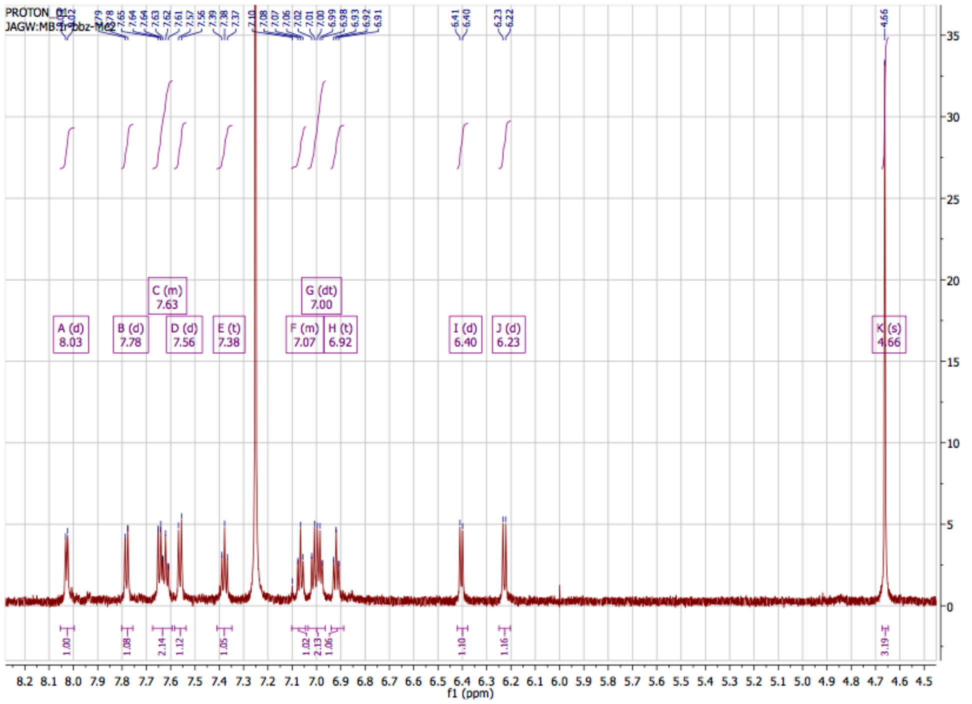

Figure S2. Absorption and emission spectra of complexes 1 and 2.

Performed in CH_2Cl_2 at 298±3 K (solid blue and red lines respectively) and emission spectra in diethyl ether / isopentane / ethanol (2:2:1 v/v) at 77 K. The absorption spectra of complexes **1** and **2** are typical of complexes of the $[Ir(N\^{}C)_2(N\^{}N)]^+$ family showing intense absorption bands with ε > 10000 M^{-1} cm^{-1} in the UV region, attributable to ligand-centred transitions, together with somewhat weaker bands extending into the visible region due to metal-to-ligand charge-transfer transitions (MLCT).[7]

Both complexes are luminescent in deoxygenated solution at room temperature, displaying broad, essentially unstructured emission, typical of emission of primarily ^{3}MLCT character. The emission quantum yields are 0.33 and 0.24 respectively, whilst the lifetimes are of the order of a microsecond, indicative of the phosphorescent nature of the emission.

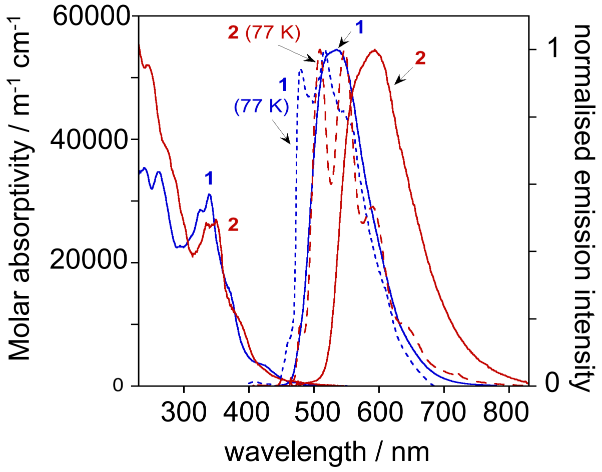

Figure S3. Two-photon emission imaging of 1 and 2 in HeLa cells. Absorption and emission spectra of complexes 1 and 2

The two-photon excitation, TPE, was performed with 800 nm light on a Zeiss LSM510 NLO Upright microscope. Left: emission images; right: brightfield images; centre: overlay of the two. Cells were incubated at 7.5 mM of each complex for 2 hours and mounted on microscope slides. Scale bars 20 mm. The data confirm the viability of the approach to TPE of complexes **1** and **2** in cells.

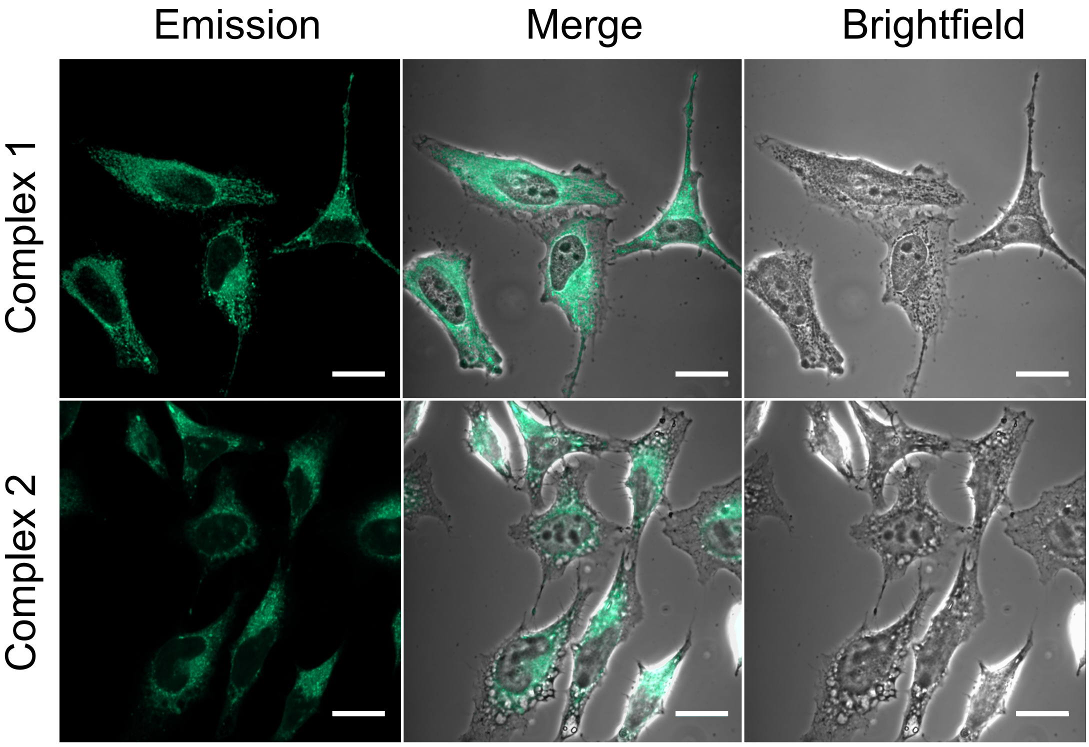
Emission
Merge
Brightfield
Complex 1
Complex 2

Figure S4. Time dependent co-localisation of complex 1 with mitochondria and lysosomes.

(A) Deconvolved wide-field images of complex 1 (green) in U2OS cells colocalised with Mitotracker orange (red left column) and Lysotracker (red right column) with central overlaid images. The cells were imaged on a Deltavision/OMX optical microscope with excitation at 405 nm and 568 nm. The 4 hour mitotracker images and the 24 hour lysotracker images are replicated in the main text. Cells were incubated with complex 1 for the times indicated then mitochondrial (mitotracker) or lysosomal (lysotracker) specific stains were added and images obtained. Scale bars = 20 mM. (B) Co-localisation indices for mitochondrial and lysosomal localization were calculated over an average of 3 images (6-9 cells/condition) using the open source imaging software Fiji (based on imagej) and the coloc 2 co-localisation tool. Values given indicate Pearson's co-efficents (r) above the threshold (the threshold regression chosen was Bisection). These co-localisation studies confirm the increased lysosomal staining over time alongside the reduced mitochondrial staining over time.

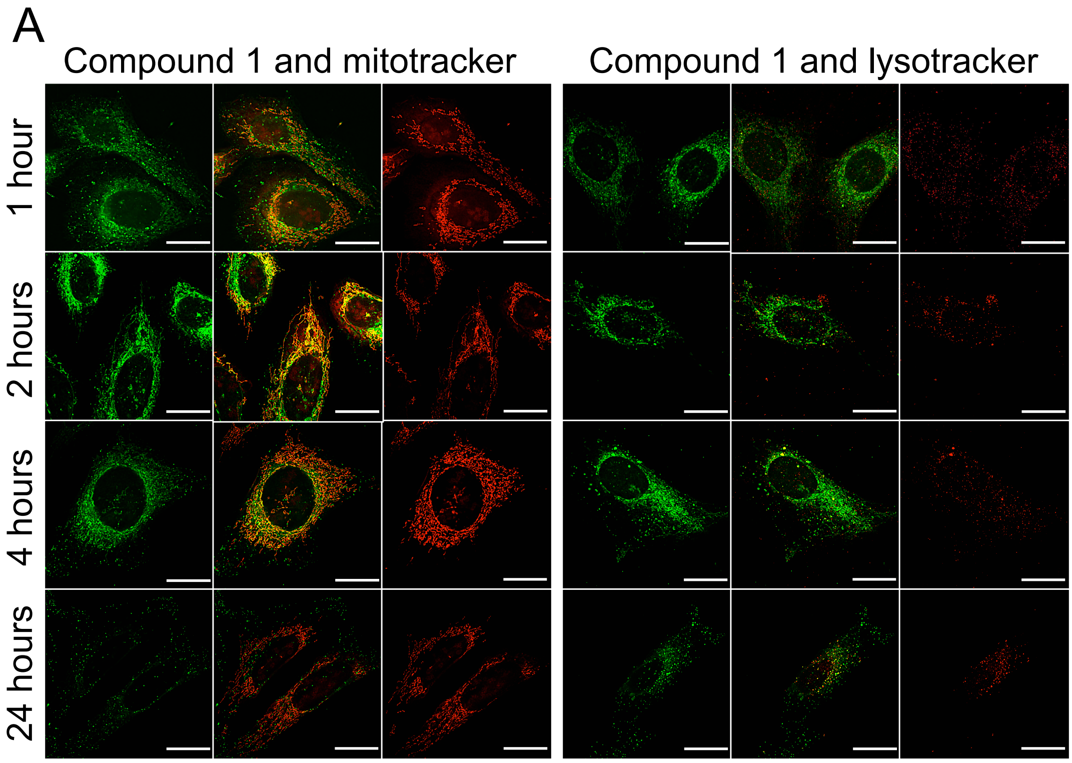

B

	Colocalisation indices (Pearson's Co-efficent, r)	
time (hours)	mitochondria	lysosomes
1	0.307	0.217
2	0.526	0.100
4	0.547	0.250
24	0.19	0.387

Figure S5. Active uptake of complex 1 by cells

(A) relative mean fluorescence intensity over 100 HeLa cells following treatment with complex **1** (5 µM) in the cold (4°C) or warm (37°C). (B) relative mean fluorescence intensity over 100 HeLa cells after treatment with complex **1** (5 µM) following preincubation with valinomycin (membrane potential modulator), chloroquine (endocytic inhibitor), tetraethylammonium (cation transport inhibitor) and DMSO (control for valinomycin). (C) repeated valinomycin result with control (DMSO), mean and standard deviation of 2 independent repeats is shown.

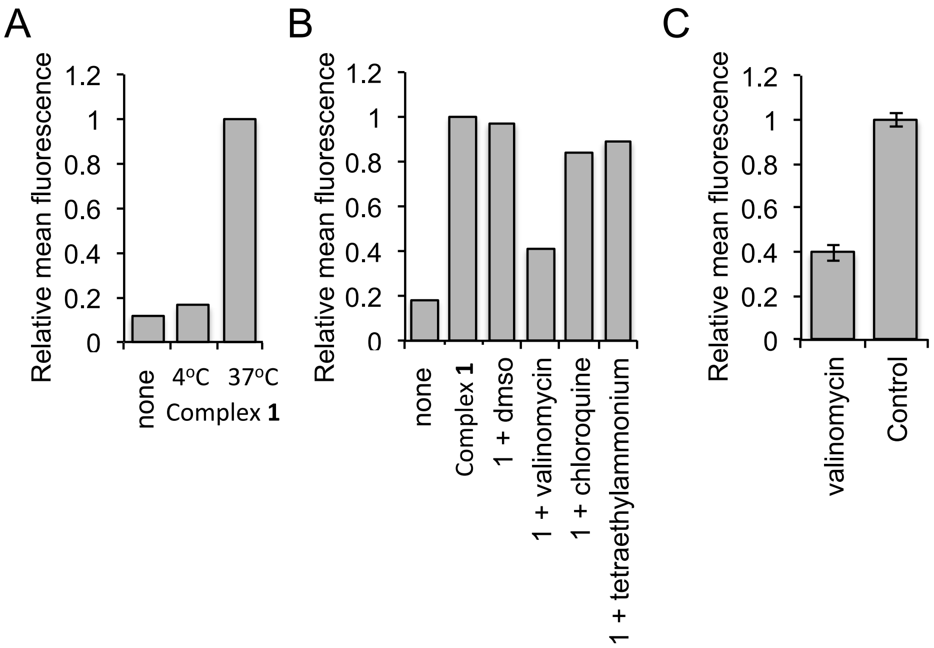

Figure S6. One-photon-induced PS activity of complexes 1 and 2.

(A) Long term MTT and clonogenic assay of cell survival following incubation of Hela cells with 100 μM complex 1. The mean and standard deviation of at least 3 independent repeats is shown. (B) LD_{50} and photoindex (PI) values for complexes 1 and 2 in various cancer cell lines. Cell death with associated apoptotic like features is also seen by microscopy where those treated with complex 1 and light are seen to become round and lift from the surface. (C) Examples of multiphoton images of Hela cells incubated with complex 1 (1 μM, 2 hours) and fixed (TOP) or (BOTTOM) pre-treated with complex 1, irradiated with light (405 nm, 20 mW, 3 minutes) and then left for 2 hours prior to fixing. Emission images (LEFT) brightfield images (RIGHT) and overlay images (MIDDLE). The two-photon excitation, TPE, was performed with 800 nm light on a Zeiss LSM510 NLO Upright microscope. Scale bars = 20 μM.

A

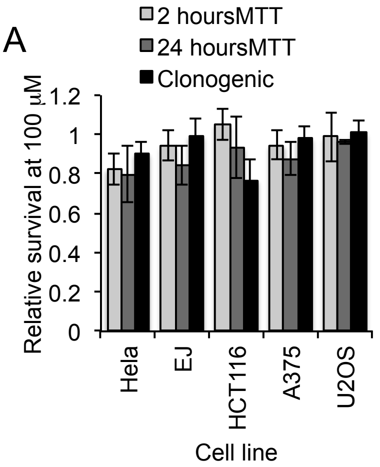

B

	Cell line	LD50 light (μM)	LD50 dark (μM)	PI
1	HeLa	0.3	>100	>333
	HCT116	0.26	>100	>384
	U2OS	0.18	>100	>555
	EJ	0.42	>100	>238
	A375	0.38	>100	>263
2	HeLa	0.5	6.5	13

C

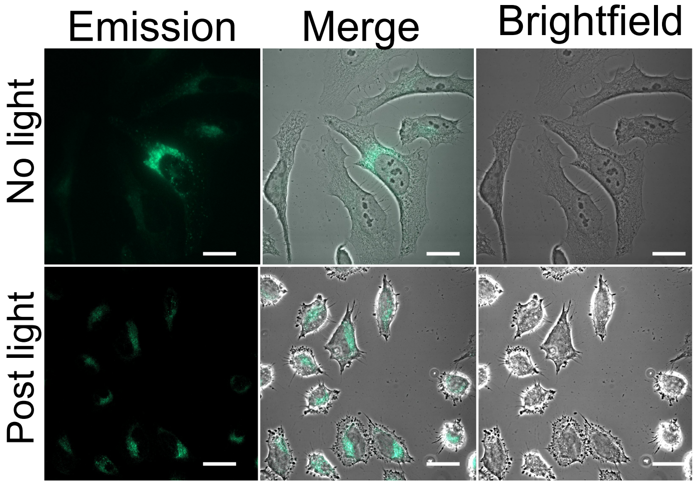

Figure S7. Light induced intracellular reactive oxygen species (ROS) generation by complex 1.

Quantification of ROS formation in HeLa cells pre-treated for 2 hours with 0.1 μM complex **1** and then exposed to 405 nm light for 3 min. Histograms generated by treatment of cells with complex **1** plus light and various controls are over laid for comparison. The fluorescence intensity on *x*-axis is proportional to the ROS levels within the cell, while cell number is indicated on *y*-axis.

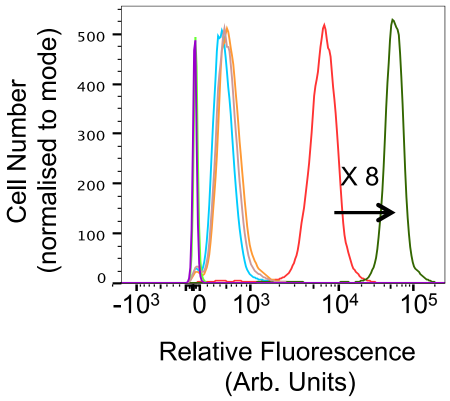

unstained all cells	no ROS detection agent
unstained live cells	
complex **1**	
no light	plus ROS detection agent
complex **1** no light	
light alone	
complex **1** plus light	

References

[1] M. Nonoyama, *Bulletin of the Chemical Society of Japan* **1974**, *47*, 767-768.

[2] N. S. Makarov, M. Drobizhev, A. Rebane, *Optics Express* **2008**, *16*, 4029-4047.

[3] K. Nakamaru, *Bulletin of the Chemical Society of Japan* **1982**, *55*, 2697-2705.

[4] aH. Huang, B. Yu, P. Zhang, J. Huang, Y. Chen, G. Gasser, L. Ji, H. Chao, *Angewandte Chemie* **2015**, *127*, 14255-14258; bH. Huang, P. Zhang, B. Yu, Y. Chen, J. Wang, L. Ji, H. Chao, *Journal of medicinal chemistry* **2014**, *57*, 8971-8983.

[5] N. M. Shavaleev, H. Adams, J. Best, R. Edge, S. Navaratnam, J. A. Weinstein, *Inorganic chemistry* **2006**, *45*, 9410-9415.

[6] R. Schmidt, C. Tanielian, R. Dunsbach, C. Wolff, *Journal of Photochemistry and Photobiology A: Chemistry* **1994**, *79*, 11-17.

[7] R. D. Costa, E. Ortí, H. J. Bolink, F. Monti, G. Accorsi, N. Armaroli, *Angewandte Chemie International Edition* **2012**, *51*, 8178-8211.
